# Developmental aspects of synaesthesia across the adult lifespan

**DOI:** 10.3389/fnhum.2014.00129

**Published:** 2014-03-11

**Authors:** Beat Meier, Nicolas Rothen, Stefan Walter

**Affiliations:** ^1^Institute of Psychology, University of BernBern, Switzerland; ^2^Center for Cognition, Learning and Memory, University of BernBern, Switzerland; ^3^Department of Psychology, Sackler Centre for Consciousness Science, University of SussexBrighton, UK

**Keywords:** consistency, color perception, age-related changes, synaesthesia attrition

## Abstract

In synaesthesia, stimuli such as sounds, words or letters trigger experiences of colors, shapes or tastes and the consistency of these experiences is a hallmark of this condition. In this study we investigate for the first time whether there are age-related changes in the consistency of synaesthetic experiences. We tested a sample of more than 400 grapheme-color synaesthetes who have color experiences when they see letters and/or digits with a well-established test of consistency. Our results showed a decline in the number of consistent grapheme-color associations across the adult lifespan. We also assessed age-related changes in the breadth of the color spectrum. The results showed that the appearance of primary colors (i.e., red, blue, and green) was mainly age-invariant. However, there was a decline in the occurrence of lurid colors while brown and achromatic tones occurred more often as concurrents in older age. These shifts in the color spectrum suggest that synaesthesia does not simply fade, but rather undergoes more comprehensive changes. We propose that these changes are the result of a combination of both age-related perceptual and memory processing shifts.

## INTRODUCTION

Synaesthesia is a relative rare variation of human experience which involves the automatic activation of an unusual concurrent sensation in response to an inducing stimulus, for example a color experience in response to a letter printed in black. The condition runs in families, thus suggesting a genetic basis, and it emerges early in development (Asher et al., 2009; Simner et al., 2009). The particular associations (i.e., inducer-concurrent pairs such as grapheme-color) are typically idiosyncratic at the individual level and stable across time. In fact, the consistency of the synaesthetic associations has been proposed as a defining characteristic of synaesthesia (Baron-Cohen et al., 1987; Cytowic and Eagleman, 2002; Rich et al., 2005; Asher et al., 2006; Simner et al., 2006). Despite this, so far no study has investigated whether consistency changes across the adult lifespan and the goal of the present study was to fill this gap.

The question whether consistency may change across the lifespan is particularly relevant for those types of synaesthesia which involve color as a concurrent, because there is clear evidence for a decrease of chromatic sensitivity in older age (Knoblauch et al., 2001; Kinnear and Sahraie, 2002; Paramei, 2012). In contrast, as synaesthesia is not a purely perceptual phenomenon, it is possible that color-associations are habitually retrieved from memory. As automatic retrieval from memory seems to be age-invariant (Meier et al., 2013) the consistency of synaesthetic perception may not be prone to age-related decline. Moreover, there is evidence that late-blind synaesthetes maintain their synaesthetic visual percepts for years after blindness, thus, synaesthesia can even persist with little or no natural sensory experience and independent from continuous associative learning (Steven and Blakemore, 2004).

In this study, we will first provide an outline that there is indeed evidence for plasticity of synaesthesia (i.e., the neonatal synaesthesia hypothesis, evidence for the development of grapheme-color synaesthesia in school age, reports of synaesthesia attrition in adolescence, variation and loss of synaesthetic experiences related to stress and brain damage). These findings motivate the present study in which we investigate changes in synaesthetic experiences over the adult lifespan, despite many studies emphasizing its stability in adult age.

According to the neonatal hypothesis of synaesthesia (Maurer and Maurer, 1988), we all may have been synaesthetes as young infants due to the increased functional connectivity in the infant brain. Synaesthesia in adults may than be viewed as a result of incomplete pruning or decreased inhibition of feedback projections in early development. While there is evidence for exuberant anatomical connectivity and for arbitrary sensory cross-activations in young infants (Huttenlocher et al., 1982; Wagner and Dobkins, 2011), and while there are early sensory cross-activations which may survive in the form of crossmodal correspondences (Spector and Maurer, 2011), the neonatal hypothesis of synaesthesia has been questioned recently (see Deroy and Spence, 2013, for a critical discussion).

In order to investigate the development of grapheme-color synaesthesia in real time within a childhood population over an extended period, Simner et al. (2009) sampled more than 600 children between 6 and 7 years. To assess consistency, the children were presented with the letters of the alphabet and the digits 0–9, one by one, in a random order on a computer screen together with an on-screen palette of 13 colors. They were required to pair each grapheme with the “best” color. After the presentation of all the graphemes, there was a short break and then the same test was repeated again. By comparing the choices of the two tests a consistency score was calculated for each child as the number of identical grapheme-color choices. On average this consistency score was 3.5 out of 36 graphemes. Next, 47 children who had scores significantly higher than this mean score were identified as potential synaesthetes and were retested in a second session 1 year later (i.e., at the age of 7/8 years). Using the same test procedure, Simner et al. (2009) identified eight children who scored highly consistent both within and across the two test sessions. These were considered as genuine synaesthetes. The other 39 children were considered as high-memory non-synaesthetes. Moreover, Simner et al. (2009) found that the number of graphemes that triggered consistent synaesthetic experiences – the bandwidth of synaesthesia (Asher et al., 2006) – increased with age. On average, the genuine synaesthetes had acquired 6.4 new grapheme-color associations over the 1-year period (i.e., they had a mean consistency score of 10.5 in Session 1 and of 16.9 in Session 2).

In a follow-up study, Simner and Bain (2013) tested these children again at age 10/11 with the same procedure as before in order to establish whether the synaesthetes performed still consistently after another 3 years – or whether synaesthesia would “die out” in some individuals which may be consistent with the neonatal hypothesis – and whether the number of consistent grapheme-color associations would further increase. Most important, the results showed that five of the eight synaesthetes identified in Simner et al. (2009) still conformed to the synaesthesia criteria. Moreover, the number of consistent graphemes for the genuine synaesthetes increased again, to 25.7 out of 36. Overall, the results of the two studies suggest that grapheme-color synaesthesia can be assesses already in 6-year old children and that the number of consistent inducer-concurrent pairs increases with development. The latter result shows that the bandwidth of the synaesthesia also increases during development (cf., Asher et al., 2006). Moreover, the results also suggest that synaesthesia can disappear during childhood, which would be consistent with the neonatal synaesthesia hypothesis.

Besides this empirical indication for the disappearance of synaesthesia in childhood, there is also evidence for synaesthesia attrition later in development. For example, there are several anecdotal reports of cases that seem to have had synaesthesia as children, but who have lost them during adolescence (Flournoy, 1893; Riggs and Karwoski, 1934; Cytowic, 1997; Emrich et al., 2000). In line with these reports, one of the authors of the present article was approached by a 25-year old student, who claimed to have had synaesthetic grapheme-color experiences as a child but had lost them during adolescence. Interestingly, she reported that she still remembered the color associations with high certainty. When tested with a synaesthetic Stroop test, she showed a reliable interference effect. The synaesthetic Stroop test involves the presentation of colored graphemes that are either congruent or incongruent to the grapheme-color association of a particular synaesthete and the participant is required to name the color of the grapheme as quickly as possible. This test has often been used to demonstrate the genuineness of synaesthesia, because synaesthetes show slower responses to incongruent compared to congruent colors, while non-synaesthetes do not show this effect (e.g., Mills et al., 1999; Odgaard et al., 1999; Dixon et al., 2004; Ward et al., 2007). However, it has been demonstrated that this effect can be induced through training grapheme-color associations in non-synaesthetes (Elias et al., 2003; Meier and Rothen, 2009; Rothen et al., 2013a; Rothen and Meier, 2014).

Further evidence for the variability of synaesthetic experiences comes from studies with adults. Rich et al. (2005) noted that “although some synaesthetes reported that the frequency or intensity of their synaesthetic experiences had diminished with age, most reported no change in their synaesthesia since childhood” (p. 67). Moreover, 28% reported that stress, alcohol, and other drugs influenced synaesthesia (either attenuating or enhancing the experience). Consistently, in a large sample of grapheme-color synaesthetes, Eagleman found similar results (in Cytowic and Eagleman, 2002, p. 139).

In an autobiographical review, Day (2013) reported that he had lost his synaesthetic experiences due to post-traumatic stress disorder (PTSD), that is, after getting into an earthquake while being trapped in the 17th floor of a shaking building with cracks running through the ceiling and the walls. He reported that although he realized this loss immediately, due to PTSD it did not start to concern him until about 2 months later. In order to revive the synaesthesia, he tried to “produce some by listening to music” (p. 918). After about 3 months, he started to worry about whether the synaesthetic experiences would come back at all, and soon later the experiences began to return very slowly, starting off very faintly and “all washed out.” It took him at least another 3 months until the synaesthetic experiences were back as before the traumatic event.

In Dittmar (2007), a woman reported the loss of her synaesthesia due to a seizure. After the incidence, she was half-sided paralyzed and also suffered from disorientation due to the changed world of experiences without synaesthesia. For her, this was particularly disturbing because she had used her synaesthesia to support her memory and planning. With rehabilitation, she was able to regain her cognitive capacity, concentration, and endurance. Concurrently, her synaesthesia came back, but not exactly as before. For example, some of her grapheme-color associations had shifted. Moreover, she reported that the intensity of the synaesthetic experience was more variable and that the experiences faded when she was under stress.

Spalding and Zangwill (1950) reported the case of a 24 year old man who was shot in the head. Besides having a large deficit in his visual memory, he reported the loss of his sequenced order of numerals and, as a consequence, innumeracy. Spalding and Zangwill supposed that the patient had lost his sequence-space synaesthesia and assumed that this was the primary cause for his disturbance.

Sacks (1997) reported the case of a painter who lost his ability to see colors after a car accident. This also resulted in a loss of his sound-color synaesthesia and he reported that listening to music had become flat and atrophied. Thus, synaesthetic experiences are variable in adult age and can even disappear forever after a brain injury.

Unfortunately, these reports did not provide detailed information about the particular brain areas that were damaged and caused the loss of synaesthesia. However, there are modern and safe techniques in neuroscience that allow the temporal suppression of specific brain areas with transcranial magnetic stimulation (TMS). For example, Esterman et al. (2006) used repetitive TMS over the posterior parietal lobes and showed that the synaesthetic Stroop effect was eliminated when applied over the right hemisphere. In contrast, TMS to the visual area V1 did not affect the synaesthetic Stroop effect. Similarly, Muggleton et al. (2007) found that single-pulse TMS over the right parieto-occipital cortex disrupted synaesthetic Stroop interference and there was also a marginally significant effect for the left parieto-occipital site, while TMS over other parietal areas showed only minimal performance disruption. Moreover, Rothen et al. (2010) showed that TMS over the parietal-occipital cortex is also effective at suppressing implicit bidirectional effects of synaesthesia.

In a recent study, we investigated whether the application of rTMS over right parietal cortex would eliminate synaesthetic experiences (Meier et al., in preparation). This question was motivated by a grapheme-color synaesthete who had explicitly expressed the wish to experience the world temporally without her synaesthesia. In order to ensure that the TMS protocol affected synaesthesia as planned we also administered the synaesthetic Stroop task before and after TMS application. We were also interested whether TMS would affect the specific grapheme-color associations and for this purpose, we administered a consistency test that required grapheme-color matches on a continuous color palette 1 week before, immediately after the TMS application and 1 week later (cf., Meier and Rothen, 2013). As expected, the results showed a synaesthetic Stroop effect before but not immediately after TMS application. However, to a big disappointment of the synaesthete, she did not experience any change in her own experience. Although she did not experience a change of her synaesthetic experiences, the consistency of the specific color experiences was reduced. That is, consistency was higher across the 2 weeks interval (i.e., 1 week before and 1 week after the TMS application) than both scores with a 1 week interval (i.e., involving the TMS application). Thus, in the latter situation, TMS seems to have influenced the synaesthetic color experiences such that she had to retrieve the specific colors from memory. Overall, the results of the TMS studies suggest that grapheme-color synaesthesia relies specifically on parieto-occipital pathways similar to normal color perception and that these regions are not only involved in explicit but also in implicit synaesthetic binding (Ramachandran and Hubbard, 2001; Hubbard and Ramachandran, 2005; Esterman et al., 2006; Rothen et al., 2010).

To summarize, there is evidence from multiple sources that the synaesthetic experiences can vary during adulthood and there are even conditions under which synaesthesia can disappear completely, either transiently or forever. There is also converging evidence from TMS-studies, as well as from functional and structural imaging studies that the occipito-parietal lobes are critically involved in grapheme-color synaesthesia (Hubbard et al., 2011; Rouw et al., 2011; Specht, 2012). To our knowledge no study has yet addressed the developmental trajectory of synaesthesia in adult age. As many cognitive functions are subject to age-related changes, it is possible that synaesthetic experiences and their consistency decline with age. However, although the occipito-parietal lobes are subject to age-related decline, this is much smaller than the decline in frontal or temporal brain areas (e.g., Brickman et al., 2006). Moreover, there is also evidence that synaesthesia can spread to novel inducers and thus it is possible that over the lifetime these opportunities accumulate (Rich et al., 2005; Mroczko et al., 2009; Blair and Berryhill, 2013; Brang et al., 2013). If so, it is possible that the bandwidth of synaesthesia may even increase across the adult lifespan. These questions are addressed in the present study. Specifically, using the method introduced by Simner et al. (2006, 2009), Rothen and Meier (2010), and Simner and Bain (2013), we investigated in a sample of more than 400 grapheme-color synaesthetes whether the number of consistent grapheme-color associations changes across the adult lifespan.

## MATERIALS AND METHODS

### PARTICIPANTS

The sample was recruited from our Synaesthesia-Check database. The Synaesthesia-Check is a short questionnaire used to establish contact with the general public interested in our research (www.synaesthesie.unibe.ch). It involves questions about potential forms of synaesthesia, the nature of synaesthetic experiences, and it provides the opportunity to leave contact information for those willing to take part in future studies. From those who had left an email address, we contacted a total of 1233 persons, who had indicated in the Synaesthesia-Check that they had consistent and involuntary synaesthetic experiences. From these, 631 persons responded to our invitation to participate in the present study. As we will focus on grapheme-color synaesthesia, we report the data of those 439 participants who indicated they had grapheme-color synaesthesia, that is, either color experiences in response to digits only (*N* = 17), letters only (*N* = 48) or both digits and letters (*N* = 374). The mean age was 38.03 years (*SD* = 13.8, range 18–91), 89.7% of them were females, and 88.4% of them were right-handed.

### PROCEDURE AND MATERIALS

**FIGURE 1:**
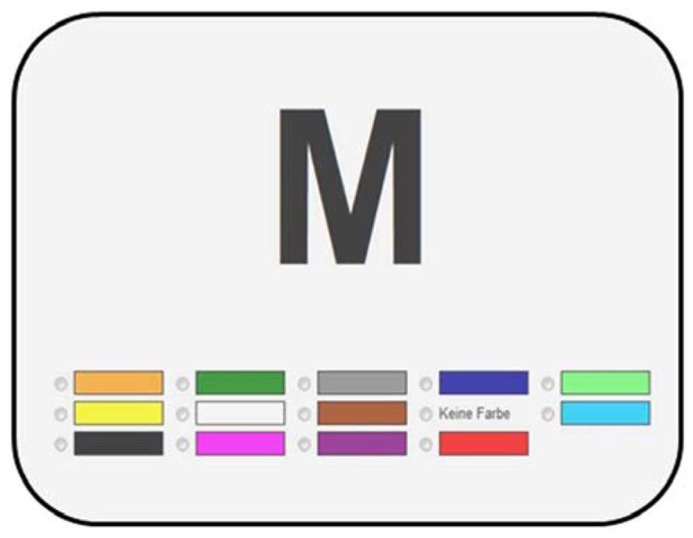
**Example trial of the consistency test**.

Participants were invited to click on a link in an email message to participate in this study which was announced as an investigation of their synaesthetic experiences. After giving consent, they were asked which forms of synaesthesia they had. Specifically, they had to indicate, separately, whether they had color experiences in response to letters, digits, words, music, and sounds. Those who had indicated color experiences to letters and digits were forwarded to a grapheme-color consistency test. The method for the consistency test was adopted from Simner et al. (2006, 2009), Rothen and Meier (2010), and Simner and Bain (2013). A computerized test individually presented 36 graphemes (A–Z; 0–9), in black on a white background, in a random order, with each presentation accompanied by a palette of 13 colors (black, dark blue, brown, dark green, gray, pink, purple, orange, red, white, light blue, light green, and yellow) and a “no color” option. The arrangement of the colors within the palette was randomized on every trial, and participants were required to select the matching color for each grapheme. An example trial is presented in **Figure 1**.

Participants were instructed to choose the color that fits best to the specific letter or digit. They were informed that if no color of the palette exactly matched their synaesthetic experience they should select the color that came closest. In the case that they had no synaesthetic color experience for a particular grapheme, they could use the “no color” option. After the presentation of all the 36 letters and digits, an immediate retest was administered in which the order of the graphemes was re-randomized.

### ANALYSIS

Digits and letters were analysed separately. As the graphemes were presented in black color on the screen, we did not consider “black” responses as synaesthetic experiences. First, the number of consistent color choices in the test and re-test were counted and a correlational analysis was performed to assess the potential relationship between age and the number of consistent colors. Next, we split the sample into three different age groups and we used analyses of variance to gain more fine-grained insights into the trajectory of the consistency scores.

In a second set of analyses, we investigated changes in the breadth of the color spectrum, separately for digits and letters. Specifically, we investigated age-related changes in the frequency in which each of the 12 colors occurred as a concurrent in each of the three age groups.

## RESULTS

### CONSISTENCY OF COLOR EXPERIENCES

**FIGURE 2:**
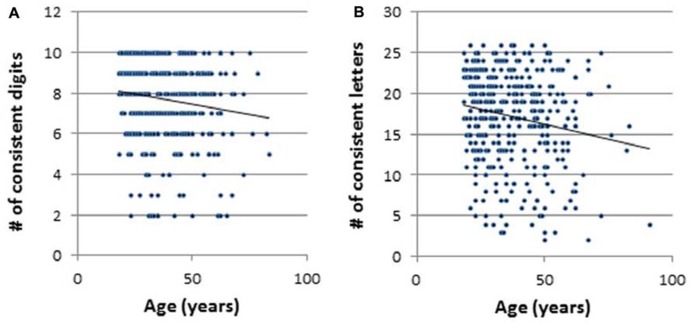
**Scatterplots of the relationship between age and the number of consistent color experiences: (A) digits, **(B)** letters.** *p < 0.05, # = number.

Scatterplots of the relationship between age and the number of consistent color experiences (consistency score, CS) are presented in **Figure 2**, separately for digits (**Figure 2A**) and for letters (**Figure 2B**). For statistical analyses alpha was set to 0.05. Correlational analyses revealed a significant negative relationship between age and the number of consistent color associations with *r* = -0.15, *p *< 0.01 for digits and *r* = -0.18, *p* < 0.01 for letters. Thus, for both digits and color there was a decrease in the number of consistent synaesthetic experiences across the lifespan.

In order to further investigate this decrease in consistency, we created three age groups (i.e., younger group aged 18–28 years, a middle group aged 29–42, and older group aged 43–91) with approximately equal sample sizes (i.e., *N* = 135, 135, and 152 for the digit-color associations and *N* = 132, 122, and 137 for letter-color associations). We considered this as a conservative approach that should eliminate spurious results due to outliers, particularly in older age.

**FIGURE 3:**
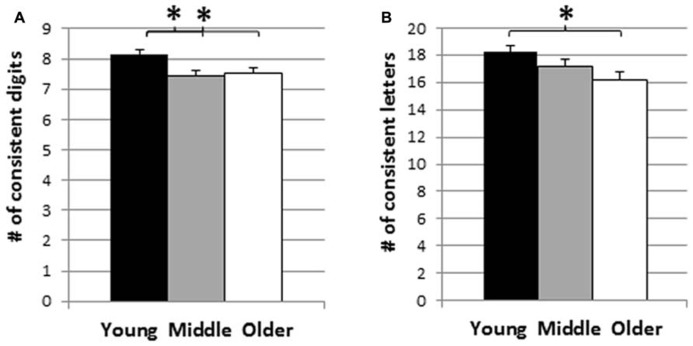
**Mean consistency scores across young (18–28 years), middle (29–42 years) and older adult age groups (43–91 years): (A) digits, **(B)** letters.** *p < 0.05, # = number.

The mean CS across the three age groups are presented in **Figure 3**, separately for digits and letters. The results of two separate one-way analyses of variance (ANOVA) showed significant age effects for both digits and letters, with *F*(2,421) = 5.80, *p* < 0.01, and *F*(2,390) = 4.26, *p* < 0.05. For digits, *post hoc* tests revealed that the consistency scores of the younger adults were higher than both the middle and older adult groups (*p*s < 0.01), while the latter two groups were not statistically different (*p* = 0.64). Similarly, for letters, *post hoc* tests revealed that the age effect was due to the higher consistency scores of the younger adults compared to the older adults (*p* < 0.01). No other effect was significant (*p*s = 0.13 and 0.18, respectively).

Next, we analysed the number of graphemes without colors (GWC), that is, those for which participants consistently chose “no color” or consistently chose the “black” color option. The GWC was *M* = 0.81 for digits (0.63, 0.98, and 1.01 for young, middle and older adults) and *M* = 3.81 for letters (2.72, 3.99, and 4.72). ANOVAs revealed an age-effect, *F*(2,421) = 6.421, and *F*(2,390) = 5.52, both *p*s < 0.01, for digits and letters, respectively. Compared to younger adults, the number of GWC was higher in older participants. For digits, *post hoc* tests showed that the young group differed from both the middle and older groups (*p*s < 0.01), while the latter did not. Similarly, for letters, *post hoc* tests showed that the young group differed from both the middle and older groups (*p*s < 0.05), and no other effect.

These findings suggest that rather than consistency *per se*, it is the bandwidth of synaesthesia that declines across the adult lifespan. Synaesthetic attrition is expressed as a reduction in the number of graphemes which trigger color experiences. To further test this interpretation, we calculated a corrected consistency score (CCS) for each participant that takes into account the number of GWC: CCS = CS/(number of possible graphemes – GWC). The number of possible graphemes is 10 for digits and 26 for letters. If our interpretation holds, then no age-effects would be expected for the CCS. The results showed that for digits, the CCS were 87.1, 82.9, and 84.3 for the young, middle, and older group. For letters, these were 78.5, 78.6, and 76.9% for the young, middle, and older group. ANOVAs failed to reach significance, * F*(2,421) = 1.83, *p* = 0.17, and *F*(2,390) < 1, *p* = 0.70, for digits and letters, respectively. Thus, these results corroborate our hypothesis that synaesthetic attrition is mainly driven by a decrease in the number of graphemes that trigger a synaesthetic color experience. If a particular grapheme triggers a synaesthetic experience, this experience is still consistent, suggesting that attrition is not due to a reduction in consistency but rather due to reduced bandwidth, thus mirroring the developmental trajectory observed in childhood (Simner and Bain, 2013).

Overall, the results from the correlational analyses and from the age-group comparisons suggest that there is a decrease in the bandwidth of synaesthetic experiences across the adult lifespan. We hypothesized that, if this interpretation is true, it is highly likely that this decrease is also reflected in the pattern of colors that constitute grapheme-color synaesthesia.

### FREQUENCY OF CONSISTENT COLORS

In a next set of analyses, we analysed how often a particular color had been chosen as a concurrent. Although this analysis was mainly exploratory, based on anecdotal reports of decreased intensity of synaesthetic color experiences and on the empirical results of age-related decline in color perception and color discrimination (Fiorentini et al., 1996; Paramei, 2012), we expected that lurid colors such as yellow, pink, or orange might be mostly affected.

In order to test whether the age-related trajectory of the 12 colors differed at all, we calculated a two-factorial ANOVA with age cohort (young, middle, older) and color (red, blue, green, yellow, orange, violet, light blue, light green, magenta, brown, silver-gray, white), separately for digits and for letters. The interaction was highly significant with *F*(22,4609) = 3.638, *p* < 0.001, for digits and with *F*(22,4268) = 3.627, *p* < 0.001, for letters, respectively, which demonstrates that the age-related trajectory differed across colors.

**FIGURE 4:**
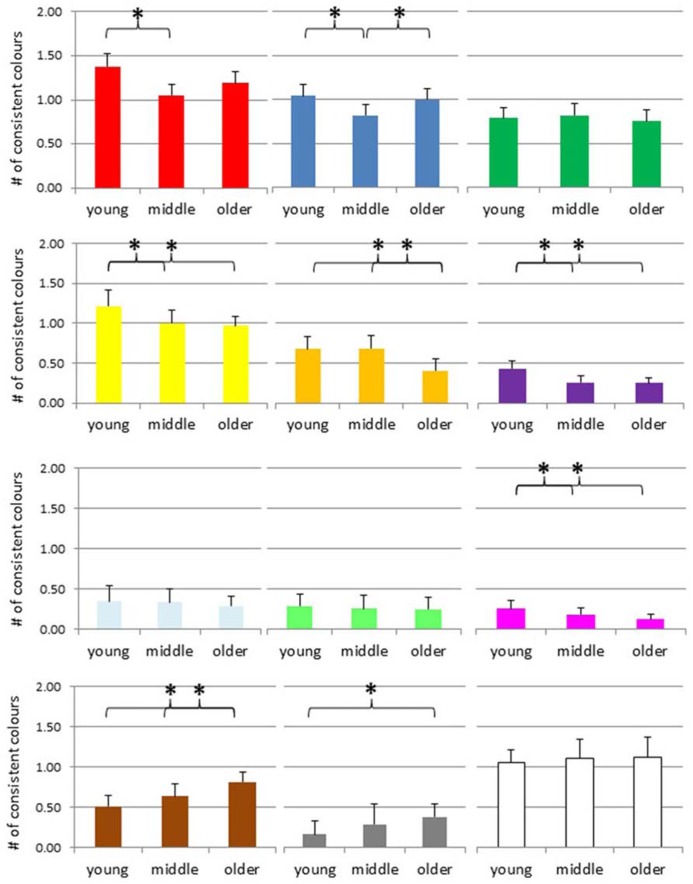
**Number of consistent colors for digits across the adult lifespan, separately for each color (red, blue, green, yellow, orange, violet, light-blue, light-green, magenta, brown, gray, and white from top left to bottom right).** *p < 0.05, # = number.

The mean number of consistent color associations for digits are presented in **Figure 4**. An ANOVA of the number of consistent *red color* experiences suggested a significant group difference, *F*(2,421) = 4.95, *p* = 0.008, and *post hoc* *t*-tests indicated that the consistency scores of the younger adults were higher than the scores of the middle adult group, *p *= 0.002; no other effect reached significance. The same kind of ANOVA for the number of consistent *blue color* experiences suggested a significant group difference, *F*(2,421) = 3.41, *p* = 0.034, and *post hoc* tests indicated that the consistency scores of the middle adults were lower than the scores of the other two groups, *p *= 0.014 compared to the young, and *p* = 0.044 compared to the older group, but no other effect reached significance, that is, no age-specific decline. The number of consistent *green color* experiences did not differ across groups, *F*(2,421) = 0.31, *p* = 0.73.

An ANOVA of the number of consistent *yellow color* experiences suggested an age-effect *F*(2,421) = 5.12, *p* = 0.006, and *post hoc* tests indicated that the consistency scores of the young group were higher than the scores of the middle and older group, *p *= 0.012 and 0.003, respectively, but no other difference. For *orange color* experiences, the ANOVA also suggested a group difference, *F*(2,421) = 4.96, *p* = 0.007, and *post hoc* tests indicated that the consistency scores of the older adults were lower than those of the two younger groups, both *p*s = 0.001, while the latter groups did not differ, *p* = 0.93. For *violet color* experiences, the ANOVA also suggested an age-related effect, *F*(2,421) = 7.73, *p* = 0.001, with *post hoc* tests indicating that the consistency scores of the young adults were higher than those of the other two groups, with *p* = 0.009 and 0.005, respectively, while the latter groups did not differ, *p* = 0.88.

Neither for *light-blue* nor for *light-green* did the ANOVA suggest a group difference, with *F*(2,421) = 0.47, *p* = 0.62, and *F*(2,421) = 0.26, *p* = 0.77. In contrast, for *magenta* the ANOVA suggested a group difference, *F*(2,421) = 3.61, *p* = 0.03, and *post hoc* tests indicated that the consistency scores of the young adults were higher than those of the older groups, *p* = 0.008, but no other difference.

In contrast, the ANOVA of the number of consistent *brown color* experiences suggested an age-related increase, *F*(2,421) = 6.94, *p* = 0.001, and *post hoc* tests indicated that the older group scored higher than the young and the middle group, *p < *0.001 and 0.031, respectively, but no other difference. For *silver-gray color* experiences, the same pattern emerged, *F*(2,421) = 5.98, *p* = 0.003, and again *post hoc* tests indicated that the consistency scores of the older adults were higher than those of the younger group, *p* < 0.001, but no other difference. For *white color* experiences, the ANOVA suggested no group difference, *F*(2,421) = 0.28, *p* = 0.76.

**FIGURE 5:**
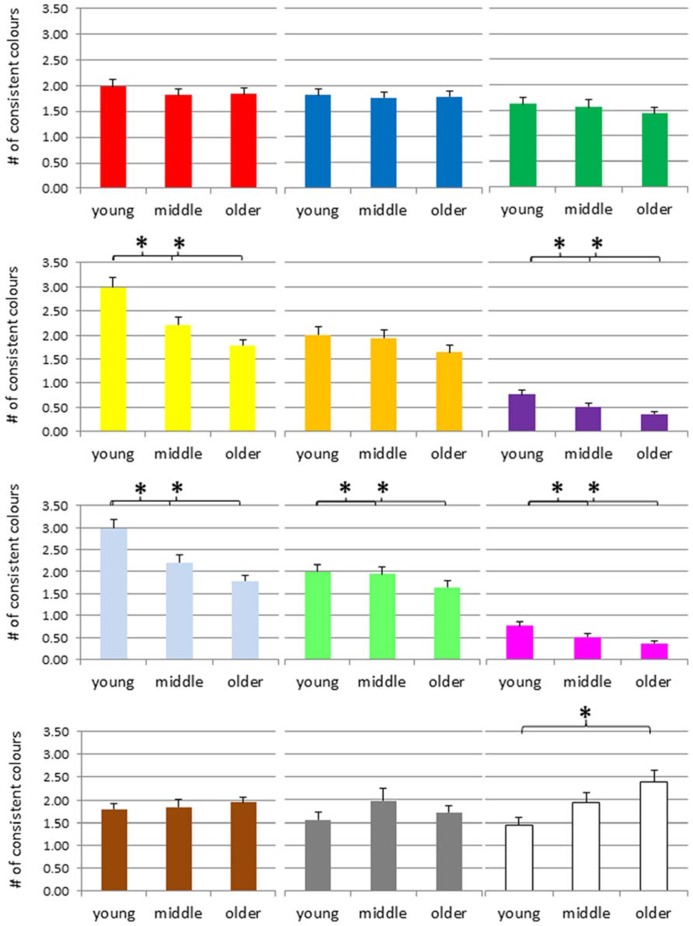
**Number of consistent colors for letters across the adult lifespan, separately for each color (red, blue, green, yellow, orange, violet, light-blue, light-green, magenta, brown, gray, and white, from top left to bottom right).** *p < 0.05, # = number.

We performed the same kind of analyses for letters. The mean number of consistent color associations for letters are presented in **Figure 5**. ANOVAs of the number of consistent *red color* experiences, *blue color* experiences, and *green color* experiences did not suggest any group differences, *F*(2,390) = 0.44, *p* = 0.64, *F*(2,390) = 0.05, *p* = 0.95, * F*(2,390) = 0.64, *p* = 0.53, respectively.

The ANOVA of the number of consistent *yellow color* experiences suggested an age-effect *F*(2,390) = 13.85, *p* < 0.001, and *post hoc* tests indicated that the young group differed from the middle and older group, *p *= 0.001 and *p* < 0.001, respectively, while the latter two groups did not, *p *= 0.077. For *orange color* experiences, the ANOVA did not suggest a group difference, *F*(2,390) = 1.63, *p* = 0.197. However, for *violet color* experiences, the ANOVA suggested a group difference, *F*(2,390) = 6.81, *p* = 0.001, and *post hoc* tests indicated that the consistency scores of the young adults were higher than those of the other two groups, with *p* = 0.03 and *p* < 0.001, respectively, while the latter groups did not differ, *p* = 0.16.

For *light-blue*, the ANOVA suggested a group difference, *F*(2,390) = 4.51, *p* = 0.012, and *post hoc* tests denoted that the consistency scores of the young adults were higher than those of the older group, *p* = 0.003, but no other effect. For *light-green,* the ANOVA also suggested a group difference, with *F*(2,390) = 3.72, *p* = 0.025, and *post hoc* tests indicated that the consistency scores of the young adults were higher than those of the other two groups, with *p* = 0.05 and 0.009, respectively, while the latter groups did not differ, *p* = 0.56. For *magenta,* the ANOVA also suggested a group difference, *F*(2,390) = 4.16, *p* = 0.016, and *post hoc* tests denoted that the consistency scores of the young adults were higher than those of the middle and older groups, with *p* = 0.03 and 0.007, respectively, while the latter groups did not differ, *p* = 0.64.

Neither for *light-blue* nor for *light-green* did the ANOVA suggest a group difference, with *F*(2,421) = 0.47, *p* = 0.62, and *F*(2,421) = 0.26, *p* = 0.77. In contrast, for *magenta* the ANOVA suggested a group difference, *F*(2,421) = 3.61, *p* = 0.03, and *post hoc* tests indicated that the consistency scores of the young adults were higher than those of the older groups, *p* = 0.008, but no other difference.

In contrast, the ANOVA of the number of consistent *brown color* experiences suggested an age-related increase, *F*(2,421) = 6.94, *p* = 0.001, and *post hoc* tests indicated that the older group scored higher than the young and the middle group, *p < *0.001, and *p* = 0.031, respectively, but no other difference. For *silver-gray color* experiences, the same pattern emerged, *F*(2,421) = 5.98, *p* = 0.003, and again *post hoc* tests indicated that the consistency scores of the older adults were higher than those of the younger group, *p* < 0.001, but no other difference. For *white color* experiences, the ANOVA suggested no group difference, *F*(2,421) = 0.28, *p* = 0.76.

We performed the same kind of analyses for letters. The mean number of consistent color associations for letters are presented in **Figure 5**. ANOVAs of the number of consistent *red color* experiences, *blue color* experiences, and *green color* experiences did not suggest any group differences, *F*(2,390) = 0.44, *p* = 0.64, *F*(2,390) = 0.05, *p* = 0.95,* F*(2,390) = 0.64, *p* = 0.53, respectively.

The ANOVA of the number of consistent *yellow color* experiences suggested an age-effect *F*(2,390) = 13.85, *p* < 0.001, and *post hoc* tests indicated that the young group differed from the middle and older group, *p *= 0.001, and *p* < 0.001, respectively, while the latter two groups did not, *p *= 0.077. For *orange color* experiences, the ANOVA did not suggest a group difference, *F*(2,390) = 1.63, *p* = 0.197. However, for *violet color* experiences, the ANOVA suggested a group difference, *F*(2,390) = 6.81, *p* = 0.001, and *post hoc* tests indicated that the consistency scores of the young adults were higher than those of the other two groups, with *p* = 0.03 and *p* < 0.001, respectively, while the latter groups did not differ, *p* = 0.16.

For *light-blue*, the ANOVA suggested a group difference, *F*(2,390) = 4.51, *p* = 0.012, and *post hoc* tests denoted that the consistency scores of the young adults were higher than those of the older group, *p* = 0.003, but no other effect. For *light-green,* the ANOVA also suggested a group difference, with *F*(2,390) = 3.72, *p* = 0.025, and *post hoc* tests indicated that the consistency scores of the young adults were higher than those of the other two groups, with *p* = 0.05 and 0.009, respectively, while the latter groups did not differ, *p* = 0.56. For *magenta,* the ANOVA also suggested a group difference, *F*(2,390) = 4.16, *p* = 0.016, and *post hoc* tests denoted that the consistency scores of the young adults were higher than those of the middle and older groups, with *p* = 0.03 and 0.007, respectively, while the latter groups did not differ, *p* = 0.64.

The ANOVA of the number of consistent *brown color* experiences suggested no age-related effect, *F*(2,390) = 0.32, *p* = 0.73. Similarly, the ANOVA of the number of consistent *silver-gray color* experiences suggested no group difference, *F*(2,390) = 1.15, *p* = 0.32. In contrast, for *white color* experiences, the ANOVA suggested an age-related increase, *F*(2,390) = 4.979, *p* = 0.007, and *post hoc* tests denoted that the consistency scores of the older adults were higher than those of the young group, *p* = 0.002, but no other effect (*p*s > 0.11).

In order to summarize the results of the age-related trajectory for individual colors, we would like to order the findings according to three distinct patterns. First, several colors seem to show an age-related decrease, such as yellow, orange, violet, light-blue or magenta. Second, there are colors that do not seem to show consistent signs of age-related changes such as red, blue or green. Finally, there seem to be several colors that rather show an increase than a decrease such as brown and white (and eventually gray, at least for digits). This pattern of results would suggest that the most frequent color terms such red, green or blue are not subject to age-related changes. In contrast, less frequent color terms such as yellow, orange, violet or magenta, which may also be considered as more intensive, seem to occur less often as synaesthetic concurrents with increasing age. Finally, white, gray, and brown, typically considered as less jaunty (or achromatic) rather seem to increase across adult age.

## DISCUSSION

This is the first study that investigated the developmental trajectory of the consistency of synaesthetic grapheme-color experiences across the adult lifespan. Specifically, using the method introduced by Simner et al. (2006), we tested a sample of more than 400 grapheme-color synaesthetes by asking them to choose the matching color for each digit and each letter of the alphabet in two separate test runs. We measured the number of consistent grapheme-color associations and assessed the relationship with age using both a correlational and a quasi-experimental approach. Our results showed a small but consistent age-related decline in the number of consistent grapheme-color associations. Together with previous results on the development of grapheme-color synaesthesia in children and adolescents in which an increase in the number of consistent grapheme color associations was found (Simner et al., 2009; Simner and Bain, 2013), the results of the present study suggest that the bandwidth of grapheme-color experiences is subject to a similar inverted u-shaped curve as many other cognitive functions (Craik and Bialystok, 2006; Zimmermann and Meier, 2006, 2010; Shing and Lindenberger, 2011; Weiermann and Meier, 2012; Meier et al., 2013).

We also assessed age-related changes in the breadth of the color spectrum. The results showed that the appearance of the most frequent color terms (i.e., red, blue, and green) was mainly age-invariant. However, lurid colors such as yellow, orange, and magenta occurred less often as synaesthetic concurrents with increasing age. In contrast, disimpassionated colors such as brown, gray, and white were chosen more often. These results underline that the age-related decline in the consistency of grapheme-color associations is not simply a chance result. They seem to be connected with systematic age-related changes in color perception and discrimination (Fiorentini et al., 1996; Knoblauch et al., 2001; Kinnear and Sahraie, 2002; Paramei, 2012).

However, the question remains why the probability declines that certain inducers trigger synaesthetic concurrents with older age. One possibility is that the graphemes do not trigger the synaesthetic experiences anymore because color perception underlies an age-related decline. However, even so, due to the lifelong associations between the inducers and the concurrent, one would expect that there are strong semantic links such that synaesthetes still would “know” the colors of the graphemes. A perceptual vs. memory distinction has been made for projector vs. associator synaesthetes (Rouw and Scholte, 2010). Accordingly, one would predict a different age-related trajectory for associate vs. projector synaesthetes. As we have not included a projector vs. associator questionnaire in this study, we cannot test this hypothesis. However, this may be an interesting avenue for future research.

Another possibility is that synaesthetic associations change across time and thus, the number of consistent grapheme color associations decreases. While we cannot exclude this possibility, this explanation cannot account for the present results because the retest was within the same test session and thus a change in associations across this short time-window is not realistic. A further explanation is that with increasing age multiple concurrents develop for a particular inducer and thus, the synaesthetic associations are systematic but not stable. For example, Simner (2012) reported that a minority of synaesthetes fulfill most of the criteria for the condition, but fail to pass the test of consistency due to changing grapheme-color associations. Although this explanation is possible, it is still not clear why multiple concurrents would be more likely in older age. Moreover, as our results suggest that the number of inducers decreases with age, this explanation cannot fully account for the whole pattern of results.

Thus, an explanation that posits that with increasing age some graphemes do not trigger the synaesthetic colors anymore* and *the specific associations are also lost is more likely. Such an explanation would imply that lower-level perceptual processes as well as higher-level cognitive processes are involved in the change of synaesthetic consistency. Specifically, it is likely that with age the processing of lurid colors is reduced such that their sensation becomes weaker and eventually fades away. Moreover, it seems also that the association between an inducer and a concurrent may change, most likely toward pastel colors, and then eventually the association is forgotten over time. For example, a yellow concurrent may fade toward white, and later on, the concurrent white experience may be completely lost. There is wide-spread evidence for an age-related decline in memory processes, and under the assumption that some of the grapheme-color associations are re-established due to fading perceptual processes, it may be that these re-established associations are also most likely to be forgotten, similar to re-consolidated memories (Nader and Hardt, 2009). However, in order to provide stronger evidence for this explanation, longitudinal data are necessary.

We acknowledge that an important restriction of the present study is its cross-sectional nature. Cross-sectional studies are not suited to investigate individual trajectories and thus rather provide a snapshot of evidence. Moreover, a further drawback of cross-sectional studies is their susceptibility to cohort effects. It may also be argued that the grapheme-color matching task draws on memory for the previously chosen association and as a consequence memory effects (potentially favoring the younger participants) may have influenced the age-related decline in the consistency measure. However, for the present study, we have no reason to assume any confounding cohort effects. Rather, we believe that by using an internet-based approach we may have biased our sample toward more active and highly motivated older adults and as such neither the decline in bandwidth nor the changes in the synaesthetic color experiences can be accounted for. Similarly, if the age-related decline in the consistency measure would be simply caused by memory effects, it would be reasonable to expect that this occurs uniformly across the color spectrum. Nevertheless, we believe that studies with a longitudinal design will lead to more detailed insights about the trajectory of synaesthesia attrition in future studies.

The main focus of this study was on the number of consistent grapheme-color associations. In line with previous studies we have used a pre-defined color palette which may not have represented the specific color experience for a particular grapheme for each synaesthete. In fact, several participants complained that the color palette was too small to capture their synaesthetic experiences. Despite this, many synaesthetes did not report synaesthetic color experiences for each and every grapheme. In fact, the age-related decline in the number of consistent grapheme-color experiences was mainly due to the fact that with increasing age fewer graphemes triggered synaesthesia. However, we would like to emphasize that the method used in the present study is not particularly suited to measure the specificity of the synaesthetic color experiences as they are used in other methods in which colors can be chosen from a much larger palette (e.g., Eagleman et al., 2007; Rothen et al., 2013b). It is an interesting direction for future research to address whether the specificity of the color experiences also changes with age. Similarly, future studies may also address the question of the intensity of the color experience more directly. So far, there is only limited evidence that the experience of color intensity changes, however, from the results of present study, we would also predict a decline in the experience of color intensity.

Rather than the specificity of synaesthetic associations, we have found that the bandwidth of grapheme-color synaesthesia was reduced across the adult lifespan. Although the bandwidth of synaesthesia is an important issue, so far, it has not received much attention. In a study on the revised test of genuineness, Asher et al. (2006) noted that broad-band synaesthetes reported percepts to a wide range of stimuli (80–100%) while narrow-band synaesthetes report percepts to a smaller range of stimuli. There are also differences in bandwidth across different types of synaesthesia. For example, in sound-color synaesthesia there is an infinite amount of inducers, while for grapheme-color synaesthesia the maximum number is clearly defined (i.e., 26 for the roman alphabet and 10 for digits). For other forms of synaesthesia the amount of possible inducers is much smaller, for example four for swimming-style synaesthesia (Nikolic et al., 2011; Rothen et al., 2013a) and only one for mirror-touch synaesthesia (see Rothen and Meier (2013) for a discussion whether the latter should be regarded as a form of synaesthesia at all). There have been suggestions that in the most common forms of synaesthesia, the bandwidth in adult synaesthetes is typically 80–100% (e.g., Ward et al., 2005; Simner and Bain, 2013). However, so far there is no clear criterion how many consistent synaesthetic grapheme-color experiences a person must have to be considered as a genuine synaesthete.

To summarize, this is the first study that has tested the synaesthesia attrition hypothesis in a large sample of grapheme-color synaesthetes. Our results showed a small but consistent decline in the bandwidth of synaesthesia across the adult lifespan and thus support the idea of synaesthesia attrition in older age. This result fits with an age-related decline in many other perceptual and cognitive functions. It also fits with evidence of an age-related decline in other forms of cross-modality (Simner and Ludwig, 2012; Ludwig and Simner, 2013). Moreover, together with recent findings from developmental studies, our results suggest that the bandwidth of synaesthesia follows an inverted u-shape function (Simner et al., 2009; Simner and Bain, 2013). We also found age-related changes in the breadth of the color spectrum of the synaesthetic concurrents. Specifically, there was a decline in the occurrence of lurid colors while brown and achromatic tones occurred more often as concurrents in older age. These shifts in the color spectrum suggest that synaesthesia does not simply fade, but rather undergoes more comprehensive changes.

## Conflict of Interest Statement

The authors declare that the research was conducted in the absence of any commercial or financial relationships that could be construed as a potential conflict of interest.
